# Immune Cell Targets of Infection at the Tick-Skin Interface during Powassan Virus Transmission

**DOI:** 10.1371/journal.pone.0155889

**Published:** 2016-05-20

**Authors:** Meghan E. Hermance, Rodrigo I. Santos, Brent C. Kelly, Gustavo Valbuena, Saravanan Thangamani

**Affiliations:** 1 Department of Pathology, University of Texas Medical Branch, Galveston, Texas, United States of America; 2 Department of Dermatology, University of Texas Medical Branch, Galveston, Texas, United States of America; 3 Institute for Human Infections and Immunity, University of Texas Medical Branch, Galveston, Texas, United States of America; 4 Center for Tropical Diseases, University of Texas Medical Branch, Galveston, Texas, United States of America; Metabiota, UNITED STATES

## Abstract

*Powassan virus* (POWV) is a tick-borne flavivirus that can result in a severe neuroinvasive disease with 50% of survivors displaying long-term neurological sequelae. Human POWV cases have been documented in Canada, the United States, and Russia. Although the number of reported POWV human cases has increased in the past fifteen years, POWV remains one of the less studied human pathogenic flaviviruses. *Ixodes* ticks are the vectors for POWV, and the virus is transmitted to a host’s skin very early during the tick feeding process. Central to the successful transmission of a tick-borne pathogen are complex interactions between the host immune response and early tick-mediated immunomodulation, all of which initially occur at the skin interface. In our prior work, we examined the cutaneous immune gene expression during the early stages of POWV-infected *Ixodes scapularis* feeding. The present study serves to further investigate the skin interface by identifying early cell targets of infection at the POWV-infected tick feeding site. An *in vivo* infection model consisting of POWV-infected ticks feeding on mice for short durations was used in this study. Skin biopsies from the tick feeding sites were harvested at various early time points, enabling us to examine the skin histopathology and detect POWV viral antigen in immune cells present at the tick feeding site. The histopathology from the present study demonstrates that neutrophil and mononuclear cell infiltrates are recruited earlier to the feeding site of a POWV-infected tick versus an uninfected tick. This is the first report demonstrating that macrophages and fibroblasts contain POWV antigens, which suggests that they are early cellular targets of infection at the tick feeding site. These data provide key insights towards defining the complex interactions between the host immune response and early tick-mediated immunomodulation.

## Introduction

*Powassan virus* (POWV) is a neuroinvasive flavivirus that is transmitted to humans from the bite of an infected tick. In 1958 POWV was first isolated from the brain tissue of a five-year-old boy who died of encephalitis in Powassan, Ontario [1]. Since then, human POWV cases have been documented in Canada, the United States, and Russia. POWV is the only North American member of the Tick-borne encephalitis serological complex of flaviviruses [2]. The most common clinical presentations of disease caused by POWV are encephalitis, meningoencephalitis, and aseptic meningitis, with an incubation period ranging from 8 to 34 days. The case fatality rate is approximately 10%, yet severe and long-lasting neurological sequelae are present in over 50% individuals who survive POW encephalitis [2]. In survivors with permanent neurological damage, recurring headaches, wasting, hemiplegia, and memory impairments are the major disease manifestations [3–6]. Although the number of reported POWV human cases has increased in the past fifteen years, POWV is one of the less studied human pathogenic flaviviruses [2]. In recent years a heightened interest in POWV has developed, likely prompted by the apparent increase in human cases and by the discovery that two separate genetic lineages of POWV exist: Lineage I which is the POWV prototype lineage and Lineage II which is the *Deer tick virus* lineage [7–10].

POWV is maintained in nature by an enzootic transmission cycle whereby *Ixodes* species ticks transmit POWV between small- to medium-sized rodents. In order for POWV to persist in nature, the ixodid tick vector must transmit the virus to a mammalian host during the tick feeding process. To successfully attach to a host and acquire a blood meal, ticks have evolved mechanisms to evade the host’s innate and adaptive immune responses. Successful tick feeding and host immune evasion is facilitated by a collection of bioactive tick salivary factors which are secreted into the feeding pool on the mammalian host’s skin. These pharmacologically active salivary components include inhibitors of the pain/itch response, anticoagulants, antiplatelet components, vasodilators, and immunomodulators [11–13]. Furthermore, the repertoire of tick salivary factors co-inoculated with a tick-borne virus can enhance viral transmission and dissemination [14–16]. When tick saliva is co-inoculated with a low dose of POWV, all mice succumb to disease and display enhanced virus dissemination and accelerated disease progression; however, mice that receive the same dose of POWV in the absence of saliva survive the infection [16]. Such findings suggest that tick saliva does more than simply serve as a vehicle for POWV transmission from the tick to a host, but instead creates a microenvironment more suitable for POWV establishment and disease development.

Although *Ixodes* species ticks will attach to a host and feed for several days, POWV is transmitted to the host very early during the tick feeding process. When a POWV-infected tick attaches to a host and initiates feeding, the virus is transmitted via tick saliva to the host’s skin within 3 hours of tick attachment [17–18]. Thus, during tick-borne virus transmission studies it is crucial to design experiments that include very early time points post tick attachment. In nature all tick-pathogen-host interactions initially occur at the cutaneous interface as the infected tick attaches to the host’s skin and begins to blood feed [12]. Skin serves as a physical barrier meant to protect the host from injury and infection. The array of skin cell populations and the many molecular mediators of inflammation are together recognized as the “skin immune system” [12,19]. As the primary line of defense between the body and environment, the skin is the first mammalian organ that POWV and tick saliva encounter on their journey from the tick salivary glands to the host’s body.

Central to the successful transmission of a tick-borne pathogen are complex interactions between the host immune response and early tick-mediated immunomodulation, all of which initially occur at the skin interface. In our recent work, we examined the cutaneous immune gene expression during the early stages of POWV-infected *Ixodes scapularis* feeding [17]. After three hours of POWV-infected tick attachment and feeding, cutaneous gene expression analysis revealed a complex pro-inflammatory environment, which included significant upregulation of genes related to granulocyte recruitment, migration, and accumulation [17]. The present study serves to further investigate the cutaneous interface during early stages of POWV-infected tick feeding. It utilizes an *in vivo* POWV infection model and provides the first report of what cell types are infected with POWV at the feeding site of a POWV-infected tick.

## Materials and Methods

### Ethics Statement

All experiments involving mice and infected ticks were conducted in arthropod containment level 3 (ACL-3) facilities in strict accordance with an animal use protocol approved by the University of Texas Medical Branch (UTMB) Institutional Animal Care and Use Committee (IACUC: # 0907054B).

### Animals

The female BALB/c mice used in this study were obtained from The Jackson Laboratory (Bar Harbor, ME). Mice were allowed to acclimate to the local environment before being incorporated into the experiments, at which point the mice were six weeks of age.

### Tick infection and infestation on mice

*I*. *scapularis* nymphal ticks were synchronously infected with POWV-LB strain as described in our previous work [17]. The infected ticks were stored inside a desiccator at 26°C to allow replication of POWV. Uninfected ticks were synchronously mock-infected with DMEM media and stored in the same manner. Four weeks post-synchronous infection the ticks were infested on mice.

Capsules for containment of tick infestation were prepared as described previously [17]. One day prior to the tick infestation, the capsules were adhered to the dorsum of mice. On the second day a single POWV-infected *I*. *scapularis* nymph was placed inside each mouse capsule and allowed to feed for 3, 6, 12, or 24 hours (N = 4 mice per time point). Uninfected *I*. *scapularis* nymphs were fed in the same manner to generate control samples. At 3, 6, 12, or 24 hours after tick attachment (hours post infection, hpi) mice were euthanized with a 350 μL intraperitoneal injection containing 10 mg/mL ketamine (Fort Dodge Animal Health, Fort Dodge, IA) and 1 mg/mL xylazine (Phoenix Pharmaceutical, St. Joseph, MO) in PBS followed by cervical dislocation. Immediately following euthanasia, 4 mm punch skin biopsies were harvested including the attached feeding tick.

### Histopathology

The skin plus attached tick biopsies were formalin-fixed for a minimum of 48 hours in 10% neutral buffered formalin. The samples were then treated with decal (Decal Chemical Corp, Tallman, NY) for approximately 2 hours and paraffin embedded. The biopsy samples were paraffin-embedded with an orientation that, upon sectioning, yielded a longitudinal section of the tick mouthpart and a cross-section of the mouse skin [20].

5-μm sections were cut from each paraffin-embedded sample, adhered to glass slides, and then deparaffinized in xylenes. Decreasing concentrations of ethanol were used to rehydrate the slides. For each paraffin-embedded skin plus tick biopsy, several sections underwent immunofluorescence (IF) staining in addition to hematoxylin-eosin (H&E) staining. H&E staining was performed following standard protocols [21], and the sections were observed under a light microscope. H&E stained tissue sections were evaluated in a randomized, blinded manner by a board-certified dermatopathologist.

The tissue sections that underwent IF staining were subjected to antigen retrieval with a citrate buffer target retrieval solution (DAKO, Carpinteria, CA) for 20 minutes with microwave heating. After returning to room temperature the sections were treated for endogenous peroxidase using BLOXALL (Vector Laboratories Inc., Burlingame, CA) for 10 minutes at room temperature. Sections were photobleached inside a UV chamber for one hour followed by a 30 minute treatment with 0.5M glycine. Antigen blocking was performed for one hour at room temperature with 5% goat serum (Sigma-Aldrich, St. Louis, MO). The following primary antibodies were incubated for 30 minutes at room temperature: rabbit anti-POWV pAb (provided by Dr. David Beasley, UTMB), chicken IgY anti-F4/80 was used for macrophage staining (antibodies-online, Atlanta, GA), and chicken IgY anti-vimentin was used for fibroblast staining (ThermoFisher Scientific, Waltham, MA). Sections were then washed and the following secondary antibodies were incubated for 30 minutes at room temperature: goat anti-rabbit IgG Alexa Fluor 647 conjugate (Life Technologies, Carlsbad, CA), goat anti-chicken IgY Alexa Fluor 546 (Life Technologies, Carlsbad, CA). All serum and antibodies were diluted in PBS with 1% BSA (ThermoFisher Scientific, Waltham, MA) and 0.1% Triton X100 (Sigma-Aldrich, St. Louis, MO), pH 7.4. Sections were counterstained with DAPI (4’,6-diamidino-2-phenylindole) (ThermoFisher Scientific, Waltham, MA) and Sudan Black B (Harleco, Gibbstown, NJ), each for 10 minutes at room temperature. The mounted and sealed slides were imaged on an Olympus BX61 fluorescence microscope using SlideBook software. To reduce natural and fixative-induced autofluorescence, the UV photobleaching and Sudan Black B steps were included [22]. The uninfected sections generated from uninfected tick feeding sites were used as negative controls to verify the specificity of the anti-POWV primary antibody. To confirm that the secondary antibodies did not bind non-specifically to cellular components, secondary antibody only (no primary antibody) controls were included.

## Results and Discussion

**Fig 1:**
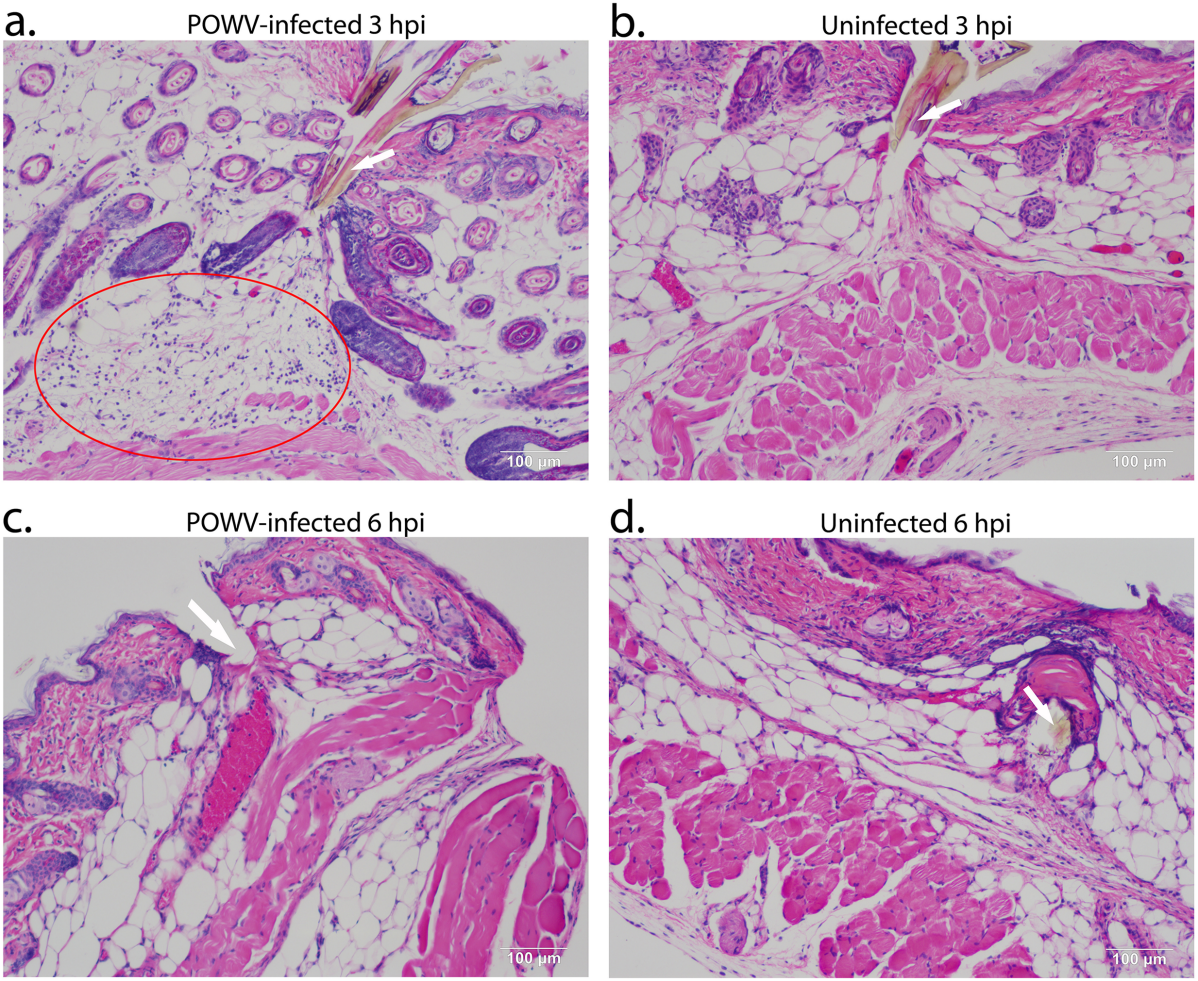
Histopathology of POWV-infected and uninfected *Ixodes scapularis* feeding sites, 3 and 6 hpi. Biopsies from tick feeding sites were sectioned and stained with H&E as described in Materials and Methods section. (**A**). POWV-infected section at 3 hpi. (**B**). Uninfected section at 3 hpi. (**C**). POWV-infected section at 6 hpi. (**D**). Uninfected section at 6 hpi. In panel **A**, the red oval indicates the area with high levels of cellular infiltrates. In panels **A**, **B**, and **D**, the arrow is pointing to the tick hypostome. In panel **C**, the arrow indicates where the tick hypostome was located prior to being dislodged during sectioning. Scale bars represent 100 μm.

**Fig 2:**
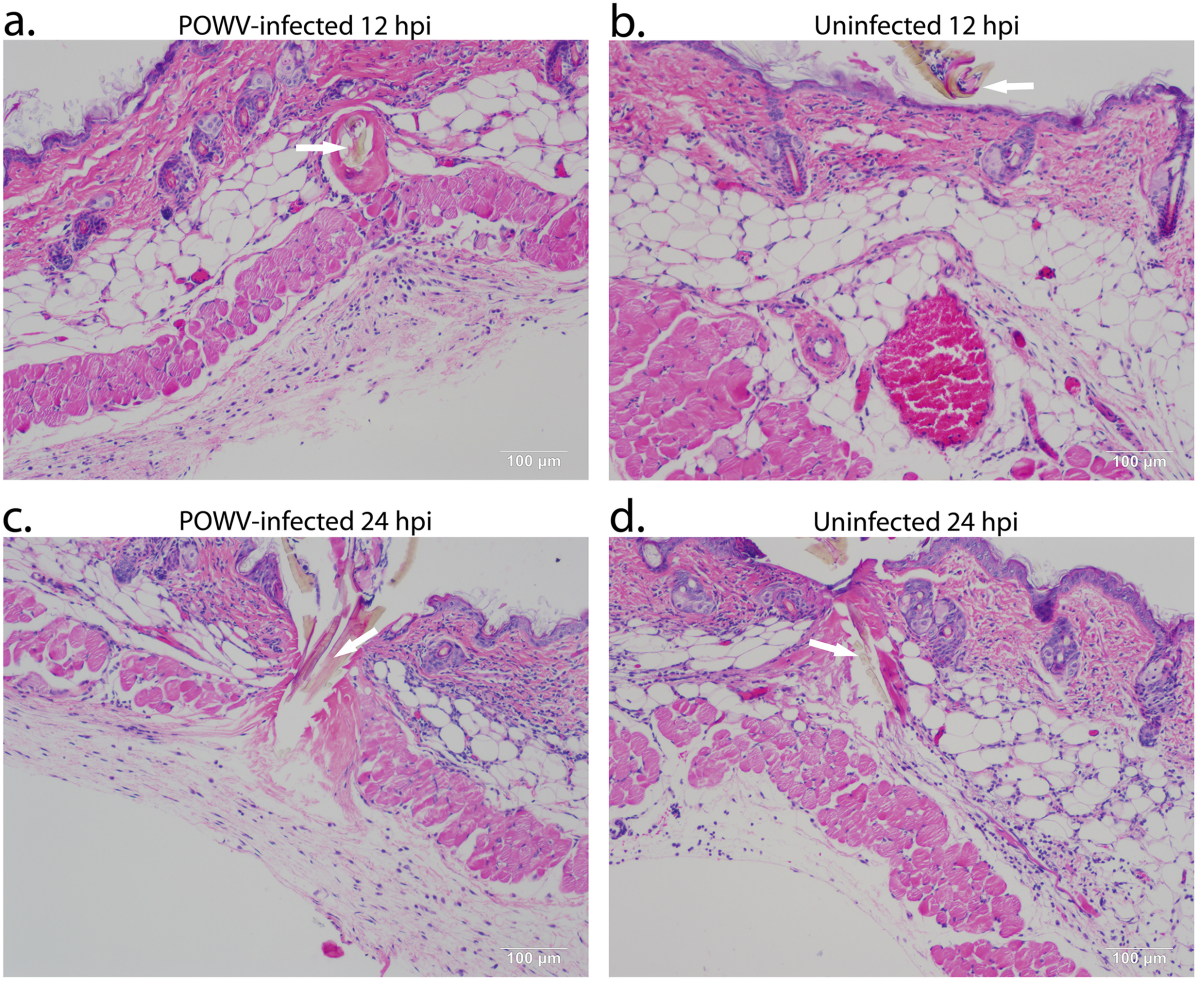
Histopathology of POWV-infected and uninfected *Ixodes scapularis* feeding sites, 12 and 24 hpi. Biopsies from tick feeding sites were sectioned and stained with H&E as described in Materials and Methods section. (**A**). POWV-infected section at 12 hpi. (**B**). Uninfected section at 12 hpi. (**C**). POWV-infected section at 24 hpi. (**D**). Uninfected section at 24 hpi. In panels **A**, **B**, **C**, and **D**, the arrow is pointing to the tick hypostome. Scale bars represent 100 μm.

To understand histopathological changes induced by the POWV-infected and uninfected tick feeding, H&E staining was performed on sections from each biopsy that most clearly showed entry of the tick hypostome into the skin. As the *I*. *scapularis* hypostome is relatively long compared to that of other tick species it was not surprising to see it penetrate through the subdermal fat cells, sometimes reaching the skeletal muscle layer (Figs 1 and 2). Human skin has a much thicker epidermis than mouse skin [23]; therefore, tick mouthparts, especially those of immature ticks, are not likely to penetrate to the subdermal muscle layer in humans. One study has compared mouse and human histology at the *I*. *scapularis* tick-skin interface [24]. Although there were a limited number of human skin biopsies in the study, the authors did not detect a significant difference between the mouse and human skin biopsies. This suggests that the immune response detected in our study is dependent on the skin immune system, not the muscle.

Near the tick mouthpart longitudinal collagen fibers appeared disrupted, demonstrating the mechanical damage caused by insertion of the serrated tick hypostome (Fig 1D). Although vascular congestion was evident, no fibrin deposition or vasculitis was observed at any time point (Figs 1 and 2).

The most distinct difference between the uninfected versus POWV-infected tick feeding sites was observed at 3 hpi. The 3 hpi POWV-infected feeding sites had higher levels of cellular infiltrates than the uninfected sites (mostly neutrophils and some mononuclear cells, Fig 1A), particularly in the deep subdermal region and extending into the skeletal muscle. This observation correlates with data from our previous study where we performed a comparative gene expression analysis between POWV-infected versus uninfected tick feeding sites at 3 and 6 hpi. The previous study demonstrated that at 3 hpi in POWV-infected tick feeding sites, several pro-inflammatory cytokines (IL1B, IL6, and IL36A) that influence the quantity of phagocytes and neutrophils during the inflammatory response were significantly upregulated [17]. The tissue sections in the present study support this finding, where the POWV-infected tick feeding sites had numerous mononuclear cell and neutrophil infiltrates at 3 hpi (Fig 1). Furthermore, at 6 hpi, both the uninfected and the POWV-infected sections had scattered neutrophil and mononuclear cell infiltrates which were less than the cellular infiltrates observed in the 3 hpi POWV-infected sections (Fig 1). This 6 hpi finding also correlates with the gene expression analysis which showed the majority of significantly modulated genes at 6 hpi to be downregulated including several proinflammatory cytokines associated with the inflammatory response reaction [17].

Our prior cutaneous gene expression analysis focused on the 3 and 6 hpi time points at the POWV-skin-tick interface; however, the present study extended the timeline to 24 hpi. At 12 hpi and 24 hpi, both the uninfected and the POWV-infected sections had mononuclear cells, neutrophils, and fibroblasts present in the sub-muscular layer (Fig 2). Lymphocytes were also present at 24 hpi in both infection conditions, but plasma cells were not found at any time point. The main difference between the early time points versus the 12 and 24 hpi time points was that enlarged fibroblasts were detected in the sub-muscular region, indicating that at 12 and 24 hpi the wound healing process was underway. Across all four time points the greatest level of cellular infiltrates was observed at the POWV-infected tick feeding site at 3 hpi.

**Fig 3:**
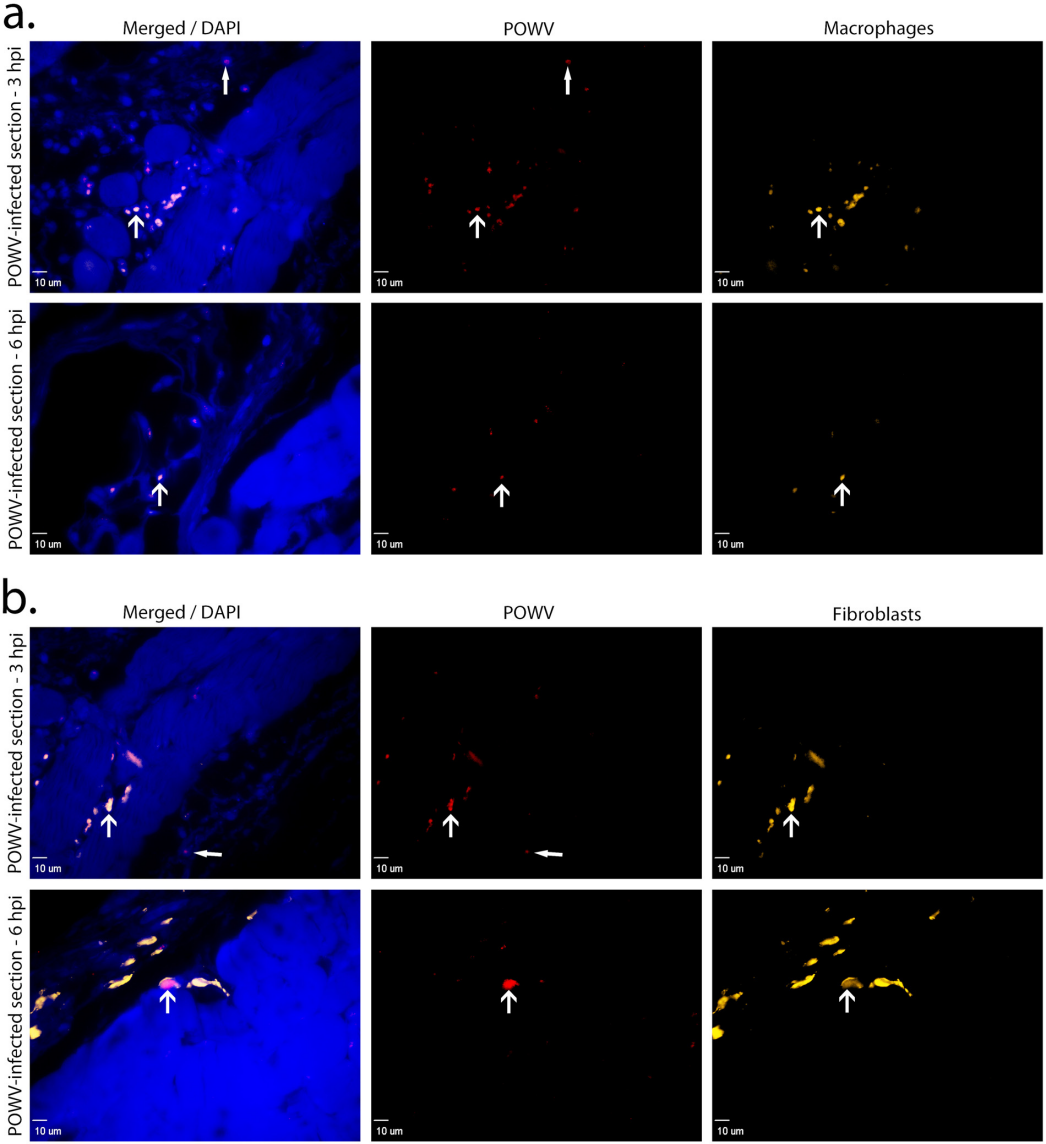
Immune cells detected at the POWV-infected *Ixodes scapularis* feeding sites, 3 and 6 hpi. (**A**). Images of skin at the POWV-infected tick feeding site where macrophages are shown in orange and POWV-infected cells are shown in red. The F4/80 marker was used for macrophage detection. (**B**). Images of skin at the POWV-infected tick feeding site where fibroblasts are shown in orange and POWV-infected cells are shown in red. The vimentin marker was used for fibroblast detection. Scale bars represent 10 μm. DAPI (4’,6-diamidino-2-phenylindole) was used for nuclear counterstaining. Open arrowheads indicate POWV-infected macrophages or POWV-infected fibroblasts. Closed arrowheads indicate other cells that are POWV-infected but that are not macrophages or fibroblasts.

**Fig 4:**
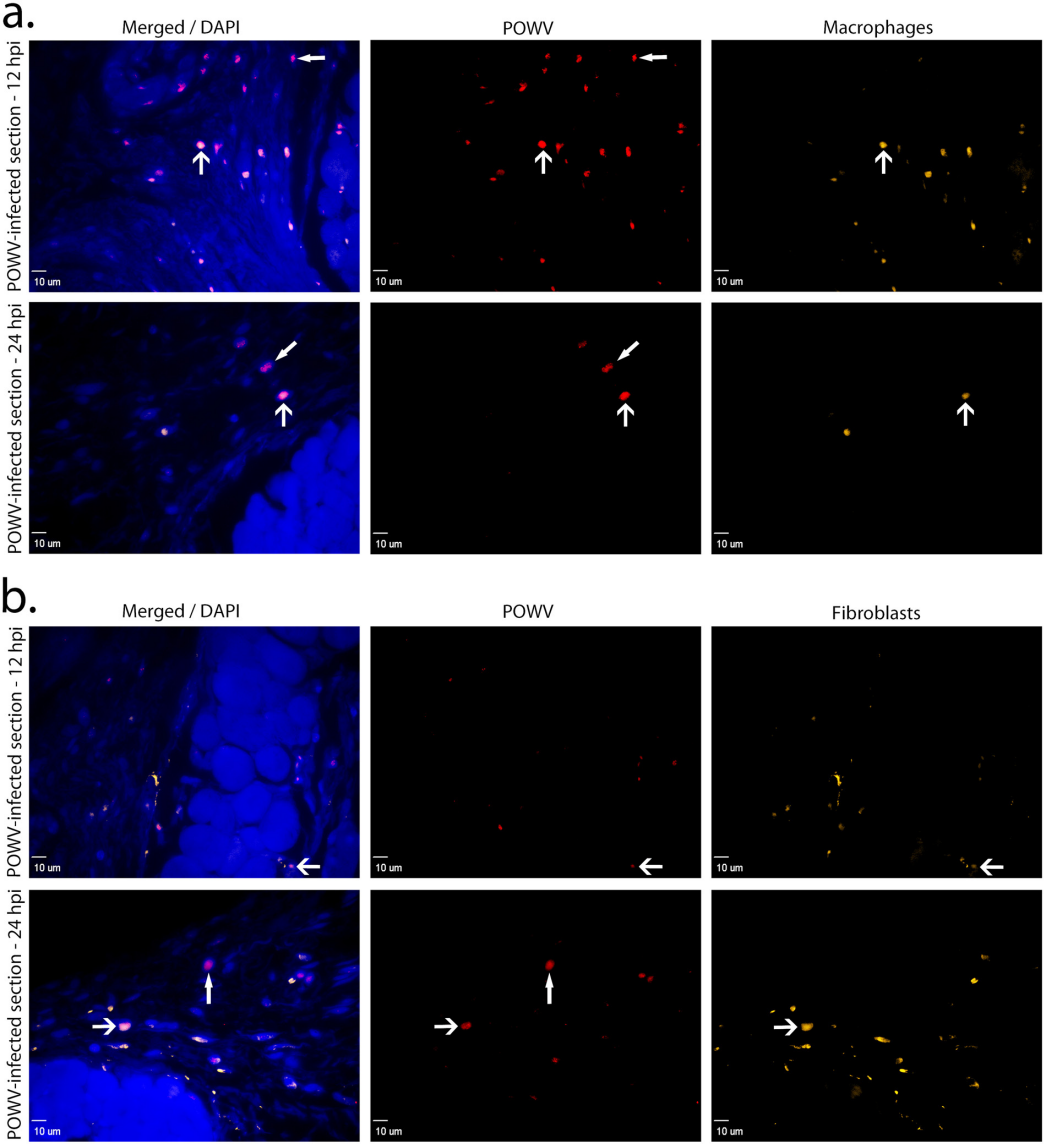
Immune cells detected at the POWV-infected *Ixodes scapularis* feeding sites, 12 and 24 hpi. (**A**). Images of skin at the POWV-infected tick feeding site where macrophages are shown in orange and POWV-infected cells are shown in red. The F4/80 marker was used for macrophage detection. (**B**). Images of skin at the POWV-infected tick feeding site where fibroblasts are shown in orange and POWV-infected cells are shown in red. The vimentin marker was used for fibroblast detection. Scale bars represent 10 μm. DAPI (4’,6-diamidino-2-phenylindole) was used for nuclear counterstaining. Open arrowheads indicate POWV-infected macrophages or POWV-infected fibroblasts. Closed arrowheads indicate immune cells that are POWV-infected but not macrophages or fibroblasts.

In parallel with the H&E analysis, we also pursued IF detection of POWV positive cells at the tick feeding site. POWV-infected cells were detected at the tick feeding site using a polyclonal antibody specific to the POWV E glycoprotein. Anti-F4/80 and anti-vimentin antibodies were used to detect macrophages and fibroblasts, respectively [25–27]. At 3 and 6 hpi, POWV was detected near the tick feeding site in macrophages and fibroblasts (Fig 3). At 12 and 24 hpi POWV-infected macrophages and fibroblasts were also detected with IF (Fig 4). As shown in Figs 3 and 4 by the closed arrowheads, other cell types that were not macrophages or fibroblasts stained positive for POWV, suggesting that POWV is capable of infecting additional cell types that were not screened for in this study.

The majority of our knowledge of arthropod-borne flavivirus transmission and pathogenesis is based on the results of needle-inoculated, relatively high doses of virus into laboratory strain animals, followed by viral load detection in various tissues. The present study is unique in that it employs a natural route of POWV delivery to the host by using infected *I*. *scapularis* ticks, a competent vector species for POWV [28]. Compared to research on POWV, more work has been conducted with other tick-borne flaviviruses such as *Langat virus* (LGTV) and *Tick-borne encephalitis virus* (TBEV). Specifically, molecular virology studies have examined the TBEV replication cycle and molecular determinants of neurovirulence and neuroinvasiveness [29]. In recent years research has begun to investigate the host immune response during TBEV and LGTV infection [30]. Prior to our gene expression analysis at the POWV-infected tick feeding site [17], no research had examined the early cutaneous immune response during tick-borne flavivirus transmission *in vivo*. The present study expands our previous work by identifying cell types that are infected with POWV at the feeding site of a POWV-infected tick, allowing us to generate a more detailed picture of the early stages of POWV transmission.

It is generally accepted that the skin is the initial infection site for arthropod-borne flaviviruses. For flaviviruses such as TBEV, LGTV and *Dengue virus*, resident dendritic cells have been shown to be among the first immune cells to express viral antigen in the skin [31–32]. Infected dendritic cells are believed to transport the flavivirus to nearby draining lymph nodes, and viral replication in these tissues leads to viremia and systemic infection [31]. Our study demonstrated that macrophages present at the tick feeding site are an early target cell for POWV infection. Although identifying dendritic cells was beyond the scope of this work, it will be an aim in our future studies as it will enable us to make further comparisons to the preceding work conducted with other arthropod-borne flaviviruses.

## Conclusions

In conclusion, this is the first report demonstrating that macrophages and fibroblasts contain POWV antigens, which suggests that they are early targets of infection at the tick feeding site. These data provide key insights towards defining the complex interactions between the host immune response and early tick-mediated immunomodulation. In our prior work, we examined the cutaneous immune gene expression during the early stages of POWV-infected tick feeding by conducting a comparative analysis between POWV-infected and uninfected tick feeding sites. In the presence of POWV-infected tick feeding, IL1B, IL6, IL36A, TLR4, and CCR3 were all significantly upregulated at 3 hpi [17]. Each of these genes helps establish the proinflammatory environment, which is generated by the increased recruitment, migration, and accumulation of immune cells. Thus, we postulated that immune cells are recruited earlier to the feeding site of a POWV-infected tick versus an uninfected tick [17]. The present study substantiates this assertion, where histopathology demonstrates that neutrophil and mononuclear cell infiltrates were recruited earlier to the feeding site of a POWV-infected tick versus an uninfected tick. Further research must be conducted to define what role, if any, macrophages and fibroblasts play in the early establishment of POWV infection. Future work will aim to determine which component of the tick-virus-host triad triggers such a rapid and early host immune response, or whether this is a cumulative effect of all components.

## Abbreviations

POWV: Powassan virus
IF: immunofluorescence
hpi: hours post infection
H&E: hematoxylin-eosin
